# Differentiated HIV service delivery model for female sex workers in sub-Saharan Africa: A systematic review

**DOI:** 10.4102/sajhivmed.v26i1.1626

**Published:** 2025-02-28

**Authors:** Lifutso Motsieloa, Edith Phalane, Amal Abdulrahman, Refilwe N. Phaswana-Mafuya

**Affiliations:** 1 South African Medical Research Council and University of Johannesburg Pan African Centre for Epidemic Research Extramural Unit, Faculty of Health Sciences, University of Johannesburg, Johannesburg, South Africa; 2 Department of Environmental Health, Faculty of Health Sciences, University of Johannesburg, Johannesburg, South Africa; 3 Key Populations Program, Center for Public Health and Human Rights, Johns Hopkins Bloomberg School of Public Health, Johns Hopkins University, Baltimore, United States of America

**Keywords:** female sex workers, key populations, differentiated service delivery model, HIV treatment outcomes, sub-Saharan Africa

## Abstract

**Background:**

Ensuring uninterrupted HIV treatment for female sex workers (FSWs), who face a disproportionately high HIV burden, is crucial for curbing HIV transmission and disease. Structural, social, and legal barriers impede their access to HIV services. The differentiated service delivery (DSD) model, designed to tailor and decentralise HIV services, aims to overcome these barriers. However, the impact of the DSD model for HIV treatment uptake among FSWs in sub-Saharan Africa (SSA) is not well documented.

**Objectives:**

To assess the implementation of the DSD model in improving HIV treatment outcomes among FSWs in SSA.

**Method:**

A systematic review literature was conducted to include available records from January 2019 to March 2024, using electronic databases such as EBSCOhost, Science Direct, SCOPUS, PubMed Central, and others. Ten studies met the eligibility criteria for inclusion for the review. The review followed the Preferred Reporting Items for Systematic Reviews and Meta-Analyses guidelines and was registered with the International Prospective Register of Systematic Reviews (ID: CRD42023440551).

**Results:**

The uptake of HIV treatment services varied depending on whether the DSD model was facility-based, community-based, or a combination of both. Community-based models were generally preferred and led to better treatment outcomes due to their comprehensive health services.

**Conclusion:**

To improve HIV treatment outcomes for FSWs in SSA, it is essential to strengthen DSD model implementation, access, and utilisation, particularly at the community level.

**What this study adds:** This systematic review has important implications for practice, and research as it enhances understanding of the barriers and facilitators of the DSD models (community, facility and mixed-model) implementation and utilisation in Sub Saharan Africa. Community-based models comprised of a comprehensive health service package which improved access to HIV treatment and outcomes among FSWs.

## Introduction

In 2023, there were 39.9 million people living with HIV (PLHIV) worldwide.^1^ While 51% of new HIV infections occurred in sub-Saharan Africa (SSA), 94% of infections occurred outside SSA.^1^ In eastern and southern Africa, young women and girls aged 15–24 years face a 2.3% higher HIV prevalence. Key populations, including female sex workers (FSWs) and their clients, men who have sex with men (MSM), people who inject drugs (PWID), and transgender people (TG) and their sexual partners accounted for 70% of the world’s HIV infections.^2^ Sex workers have a 3% higher rate of HIV prevalence.^1^ FSWs often engage in unprotected sex and transactional sexual activity with several partners, and account for 40% of the HIV prevalence in SSA.^3^ Moreover, the estimated HIV prevalence among the FSW population is 10- to 20-fold greater than the general population, notwithstanding significant geographical variance.^4^ For instance, in Rwanda, 50% of FSWs are living with HIV.^4^ In Uganda, HIV prevalence among FSW is 33% – 37%, compared to 9.5% among the general female population.^5^

The risk of HIV acquisition among key populations, especially FSWs, requires an understanding in the context of economic hardship, violence, and criminalisation of sex work.^3^ The standard care of delivery is associated with unmet needs of HIV services for FSWs.^6^ It involves a uniform approach to healthcare, where all patients follow the same care of HIV treatment, regardless of their unique needs or circumstances.^7^ This model typically requires patients to visit healthcare facilities frequently for clinical check-ups, medication collection, and ongoing care.^8,9^ The services for HIV are centralised, delivered by healthcare providers in structured clinical environments, offering limited flexibility in terms of scheduling.^9^ As a result, FSWs may face challenges such as long waiting periods, inflexible hours, stigma, and inability to access consistent HIV care.^6,7^ As a result, in ensuring that there is uninterrupted delivery of HIV treatment services to curb transmissions and new infections amongst key populations, the differentiated service delivery (DSD) model was introduced to address barriers towards treatment access for continuity of services.^10^

The DSD model provides an alternative to the standard care of HIV treatment delivery, which has historically been provided in fixed-site clinics and necessitates at least four patient visits to the clinic per year, if not more.^11^ This model is designed to be patient-centred and to enhance the efficiency of health systems by adapting HIV care to meet patients’ needs and the capacities of health services.^6,9^ Unlike the standard approach, which applies uniform care to all patients, the DSD model tailors HIV services based on a patient’s antiretroviral therapy (ART) stability, reducing clinic visits and offering decentralised care.^9^ While the DSD model has shown positive effects on HIV outcomes in SSA in the general population, its specific benefits for FSWs remain less evident.^12^ FSWs face distinct challenges, such as social, structural, and legal barriers, which require more focused research to understand how the DSD model impacts their HIV treatment outcomes.^13^

The DSD models include community-based, facility-based and mixed models. Community-based models, such as community ART groups (CAGs), facilitate group medication collection and drug distribution by community health workers.^6^ Task-shifting empowers community health workers to deliver specific services, improving access and reducing healthcare burdens.^14^ The facility-based model provides quick medication access and extended appointment intervals for persons that are adherent to ART. Additionally, peer-led programmes offer support and education,^9^ while mobile clinics and outreach services extend care to underserved communities.^15^ Family-oriented care integrates HIV services with maternal and child health.^6,15^ The purpose of this article is to assess the implementation of the DSD model in improving HIV treatment outcomes among FSWs in SSA.

## Research methods and design

### Approach

The systematic review adhered to the Preferred Reporting Items for Systematic Reviews and Meta-Analysis (PRISMA) guidelines to ensure validity and reliability (see Online Appendix 1 for the PRISMA Checklist).^16^ The review was registered on the International Prospective Register of Systematic Reviews (ID: CRD42023440551).

### Search strategy

The search strategy was developed using the Population, Intervention, Comparison, and Outcome (PICO) framework, Boolean operators (‘AND’ and ‘OR’), truncation (‘*’ and ‘()’), and Medical Subject Heading terms. The systematic review includes peer-reviewed published and full-text-accessible articles of primary studies conducted in SSA on implementation of the DSD model to enhance HIV treatment among FSWs. A combination of key concepts (main key terms and their synonyms) was used. In searching, the Boolean operator ‘OR’ was used to link each key concept to their synonyms, and ‘AND’ to combine main key terms, while the truncations were used to separate or emphasise the synonyms and main key terms. The search terms and strategy were combined in the manner presented in Table 1.

**TABLE 1:** Search strategy.

Domain	Search strategy
[#1] DSD	‘Differentiated Service Delivery Model’ OR ‘Fast Track’ OR ‘HIV Patient Centred Care’ OR ‘Community Clubs’ OR ‘Pharmacy Pickups’ OR ‘client centred approach’ OR ‘CCMD’ OR ‘Decanting’
[#2] FSW	‘Female Sex Workers Living with HIV’ OR ‘FSW’ OR ‘SW’ OR ‘FSWPLHIV’
[#3] HIV OUTCOMES	‘HIV Clinical Outcomes’ OR ‘Viral Suppression’ OR ‘Viral Load Suppression’ OR ‘Virologic Outcomes’ OR ‘Treatment outcome’ OR ‘Linkage to care’ OR ‘95 95 95 strategy’
[#4] SSA	‘Sub-Saharan Africa*’ OR ‘sub-Saharan’ OR ‘sub-Sahara Africa’ OR SSA* OR ‘Angola’ OR ‘Benin’ OR ‘Botswana’ OR ‘Burkina Faso’ OR ‘Burundi’ OR ‘Cabo Verde (Cape Verde)’ OR ‘Cameroon’ OR ‘Central African Republic’ OR ‘Chad’ OR ‘Comoros’ OR ‘Congo (Brazzaville)’ OR ‘Democratic Republic of the Congo (Kinshasa)’ OR ‘Djibouti’ OR ‘Equatorial’ ‘Guinea’ OR ‘Eritrea’, ‘Eswatini (formerly Swaziland)’ OR ‘Ethiopia’ OR ‘Gabon’ OR ‘The Gambia’ OR ‘Ghana’ OR ‘Guinea’ OR ‘Guinea-Bissau’ OR ‘Ivory Coast (Côte d’Ivoire)’ OR ‘Kenya’ OR ‘Lesotho’ OR ‘Liberia’ OR ‘Madagascar’ OR ‘Malawi’ OR ‘Mali’ OR ‘Mauritania’ OR ‘Mauritius’ OR ‘Mozambique’ OR ‘Namibia’ OR ‘Niger’ OR ‘Nigeria’ OR ‘Rwanda’ OR ‘Sao Tome’ and ‘Principe’ OR ‘Senegal’ OR ‘Seychelles’ OR ‘Sierra Leone’ OR ‘Somalia’ OR ‘South Africa’ OR ‘South Sudan’ OR ‘Sudan’ OR ‘Tanzania’ OR ‘Togo’ OR ‘Uganda’ OR’ Zambia’ OR ‘Zimbabwe’
Combination of 1–4	‘Differentiated Service Delivery Model’ OR ‘Fast Track’ OR ‘HIV Patient Centred Care’ OR Community Clubs’ OR Pharmacy Pickups’ OR ‘client centred approach’ OR ‘CCMD’ OR ‘Decanting’ AND ‘Female Sex Workers Living with HIV’ OR ‘FSW’ OR ‘SW’ OR ‘FSWPLHIV’ AND ‘HIV Clinical Outcomes’ OR ‘Viral Suppression’ OR ‘Viral Load Suppression’ OR ‘Virologic Outcomes’ OR ‘Treatment outcome’ OR ‘Linkage to care’ OR ‘95 95 95 strategy’ AND ‘‘Sub-Saharan Africa*’ OR ‘sub-Saharan OR sub-Sahara Africa’ OR ‘SSA’* OR ‘Angola’ OR ‘Benin’ OR ‘Botswana’ OR ‘Burkina Faso’ OR ‘Burundi’ OR ‘Cabo Verde (Cape Verde)’ OR ‘Cameroon’ OR ‘Central African Republic’ OR ‘Chad’ OR ‘Comoros’ OR ‘Congo (Brazzaville)’ OR ‘Democratic Republic of the Congo (Kinshasa)’ OR ‘Djibouti’ OR ‘Equatorial Guinea’ OR ‘Eritrea’ ‘Eswatini (formerly Swaziland)’ OR ‘Ethiopia’ OR ‘Gabon’ OR ‘The Gambia’ OR ‘Ghana’ OR ‘Guinea’ OR ‘Guinea-Bissau’ OR ‘Ivory Coast (Côte d’Ivoire)’ OR ‘Kenya’ OR ‘Lesotho’ OR ‘Liberia’ OR ‘Madagascar’ OR ‘Malawi’ OR ‘Mali’ OR ‘Mauritania’ OR ‘Mauritius’ OR ‘Mozambique’ OR ‘Namibia’ OR ‘Niger’ OR ‘Nigeria’ OR ‘Rwanda’ OR ‘Sao Tome’ and ‘Principe’ OR ‘Senegal’ OR ‘Seychelles’ OR ‘Sierra Leone’ OR ‘Somalia’ OR ‘South Africa’ OR ‘South Sudan’ OR ‘Sudan’ OR ‘Tanzania’ OR ‘Togo’ OR ‘Uganda’ OR ‘Zambia’ OR ‘Zimbabwe’
ADD	(**#1**); (**#1 & #2**); (**#1 & #2 & #3**); (**#1 & #2 & #3 & #4**); (**#1 & #2)**

CCMD, Central Chronic Medicines Dispensing; DSD, differentiated service delivery; FSWPLHIV; Female Sex Workers Living with HIV; FSW, female sex worker; SSA, sub-Saharan Africa; SW, sex worker.

The electronic databases included EBSCOhost, Science Direct, SCOPUS, PubMed Central, PLOS Global Public Health, PLoS ONE, Crossref, Medline, Google Scholar, and Lancet. Additional information was sought from multilateral organisations such as the Joint United Nations Programme on HIV/AIDS, World Bank, US Centers for Disease Control and Prevention, and Global Fund. Reports and unpublished articles from these organisations were also reviewed. In addition, the reference list of selected studies was also consulted to ensure literature saturation.

### Eligibility criteria

The PICO framework was used to guide and establish the eligibility criteria. The key elements of the PICO framework were used to define and structure the inclusion and exclusion criteria (see Table 2). The search strategy included HIV treatment outcomes. The search was done to include peer-reviewed articles published between the years 2019 and 2024 to understand the impact of the DSD model on HIV treatment outcomes among FSWs in SSA. The review included studies reporting on FSWs on ART aged 15 years and older, as there is growing evidence demonstrating that adolescent girls and young women report being involved in transactional sex.^17^

**TABLE 2:** Inclusion and exclusion criteria of the literature for the systematic review.

Aspect	Inclusion criteria	Exclusion criteria
Population	Studies reporting on: FSW on ART (Cisgender), irrespective of race FSW aged 15 years and above Sub-Saharan Africa	Studies that are not reporting on FSWs Studies done outside sub-Saharan Africa Studies reporting on FSW not on ART
Intervention	Delivery of ART/HIV treatment for FSWs living with HIV through the DSD model, implemented either at healthcare facilities or at the community level, aims to improve HIV treatment outcomes Required descriptive data about the model: *When: frequency of service delivery; Where: place of service delivery; Who: person providing the service and What; services offered*	Any intervention not related to ART delivery or HIV treatment through the DSD model
Study comparison	Not required – single-arm evaluations are eligible	Not relevant to the study
Outcomes	**HIV treatment outcomes** Viral load suppression Viral suppression Virologic outcomes Antiretroviral uptake Antiretroviral adherence 95-95-95 Linkage to care **DSD implementation outcomes** Sustainability Acceptability Feasibility	Not describing DSD treatment outcomes Lost to follow-up Defaulter Viral load not done CD4 not done
Study design	Cohort studies Observational studies Cross-sectional studies Prospective studies Qualitative studies Exploratory studies Case-control studies Randomised controlled trials Non-randomised controlled trials	Meta-analyses, systematic reviews, scoping reviews Non-HIV DSD model focus Commentaries Editorials Modelling studies Case reports Treatment guidelines Publication that did not report primary data.

ART, antiretroviral therapy; DSD, differentiated service delivery; FSWs, female sex workers.

### Study selection

Endnote and Covidence tools were used to remove any duplicates and manage the screening process using the purposely designed eligibility criteria mentioned above. The tools were also used to store and organise the references. Following the removal of duplicate articles, two reviewers (L.M. and A.A.) independently screened titles, abstracts, and full text to exclude articles that did not meet the inclusion criteria. The third (E.P.) and fourth (R.N.P.-M.) reviewers supervised the selection process through periodic checks to ensure the quality of the process. Disagreements were resolved by the team through consensus. The team held meetings to resolve conflicts using the Covidence software tool. The quality and bias of the included articles were assessed utilising the Critical Appraisal Skills Programme (CASP) tool in Online Appendix 2.

### Data extraction

Ten studies included in the review were extracted in accordance with the aims of this review by utilising the data extraction tool in Covidence.^18^ Two authors (L.M. and A.A.) independently reviewed and extracted each article before jointly reviewing the extracted data to reach a consensus. The literature reporting on the use of DSD models for HIV treatment among FSWs were included. Studies that reported on HIV treatment implementation, ART service delivery, retention to care, and viral load testing coverage were also considered. The information extracted was synthesised based on relevant information on study characteristics, the DSD model type, purpose of the DSD model, challenges, and success in utilising the DSD model for HIV treatment.

### Quality assessment

The qualitative studies were assessed utilising the CASP tool to verify the quality of the selected articles and to screen them for bias (see Online Appendix 2).^19^ The quality assessment tool for observational cohort and cross-sectional studies (see Online Appendix 3)^20^ was used to assess the cross-sectional studies. Two reviewers (L.M. and A.A.) independently evaluated each article before jointly verifying the results.

### Review selection

**FIGURE 1:**
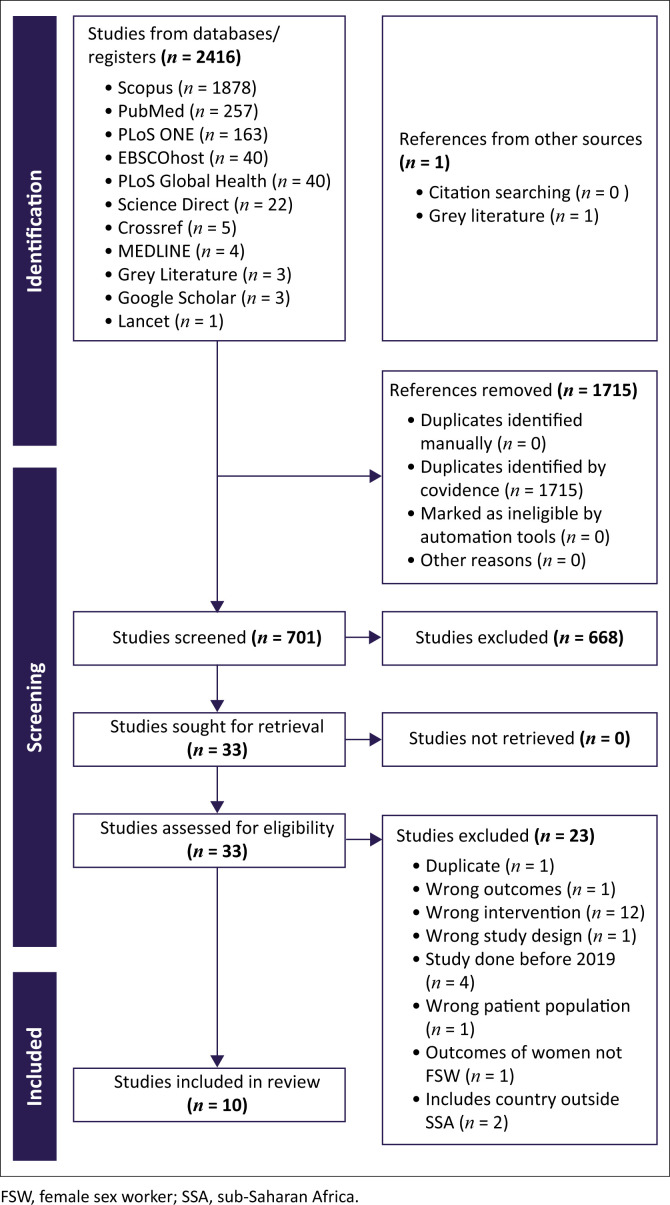
Preferred reporting items for systematic reviews and meta-analysis.

The identification, screening, eligibility, and inclusion are summarised in Figure 1. There were 2416 studies identified, including one grey literature study, and 1715 of the 2416 studies were removed as duplicates. The remaining 701 articles were screened for title and abstract, and 668 articles were excluded as they did not meet the eligibility criteria stated in Table 2 and Figure 1. As a result, 10 eligible articles were identified and included in the study.

### Ethical considerations

As no original data were collected, ethical approval and consent for participation were not sought for this review. However, this review is part of the doctoral research undertaken by Lifutso Motsieloa (L.M.), which has received approval from UJ Faculty of Health Science Research Ethics Committee (reference no.: REC-2519-2024).

## Results

The review included an initial search that identified 2416 records, including one grey literature study, and 1715 duplicates were identified by Covidence. A total of 701 articles and one grey literature study were included in the abstract and title screening stage. After screening for relevance, 668 records were excluded based on abstract and title screening. Subsequently, 33 records qualified for full text review and 23 studies were excluded because they fell outside the study criteria due to various reasons including wrong outcome, wrong intervention, wrong study design, wrong population, and study conducted published earlier than 2019. Ten published studies and grey literature were included in data extraction as they met the inclusion criteria for the analysis.

### Study characteristics

The 10 eligible articles (nine published and one grey literature) included in the review are from five SSA countries, namely South Africa, Tanzania, Uganda, Zimbabwe, and Malawi. Table 3 presents the characteristics of the eligible articles. These articles explored a range of topics on the implementation of the DSD model, quality of care, enrolment and retention in HIV care, factors associated with retention and non-viral suppression, experiences of accessing HIV care services, and ART. The majority of the studies included were qualitative studies (*n* = 6), followed by programme/routine, cross-sectional study/grey literature report (*n* = 2), and prospective cohort design (*n* = 2). The sample size of the studies ranged from *n* = 20 to *n* = 617 and included FSWs aged 18 years or older. A few studies also included additional populations such as healthcare workers (HCWs) and key informants.

**TABLE 3:** Summary of study characteristics.

Authors, year	Reference no.	Title	Locality	Country	Data type, study design	Study population	Sample size	Study period
Tun et al., 2023	22	Quality of care is perceived to be high with community-based antiretroviral therapy (ART) services for female sex workers in Tanzania: Qualitative findings from a pilot implementation science study	Njombe and Mbeya	Tanzania	Research, Qualitative study	FSW living with HIV aged 18+ years	24 total: 12 intervention and 12 comparison	July 2017 – October 2017
Arinaitwe et al., 2023	24	Enrollment and retention of female sex workers in HIV care in health facilities in Mbarara city	Mbarara City, Southwestern Uganda	Uganda	Research, Qualitative study	FSW living with HIV; HCWs aged 21–50 years	51 total: 30 FSW and 21 HCW	February 2022 – April 2022
Comins et al., 2022	29	Opportunities and considerations for the design of decentralized delivery of antiretroviral therapy for female sex workers living with HIV in South Africa	Durban	South Africa	Research, Qualitative study	FSW, key informants aged 18–50 years	39 total: 24 FSW and 15 key Informants	September 2017 – November 2017
Atuhaire et al., 2022	25	A retrospective cross-sectional study assessing factors associated with retention and non-viral suppression among HIV positive FSWs receiving antiretroviral therapy from primary healthcare facilities in Kampala, Uganda	Kampala	Uganda	Programme/ Routine, Cross-sectional study	FSW living with HIV initiating ART aged 25–35 years	275	January 2018 – December 2020
Atuhaire et al., 2022	26	‘My condition is my secret’: perspectives of HIV positive female sex workers on differentiated service delivery models in Kampala Uganda	Kampala	Uganda	Research, Qualitative study	FSW living with HIV aged 20–50 years	24	January 2021
Moyo et al., 2021	28	The experiences of sex workers accessing HIV care services in Bulawayo, Zimbabwe	Bulawayo	Zimbabwe	Research, Qualitative study	HIV+ FSW living with HIV aged 25–45 years	20	December 2018 – March 2019
Vu et al., 2021	27	Assessment of community-based ART service model linking female sex workers to HIV care and treatment in Blantyre and Mangochi, Malawi	Blantyre and Mangochi	Malawi	Mixed-method implementation science study (Prospective cohort design)	FSWs ART-naïve or those who previously defaulted from ART for at least 90 days – median age was 25 years old	194	June 2018 – August 2018
Nyato et al., 2019	23	Facilitators and barriers to linkage to HIV care and treatment among female sex workers in a community-based HIV prevention intervention in Tanzania: A qualitative study	Dar es Salaam: Mbeya, Iringa, and Shinyanga	Tanzania	Research, Qualitative study	FSW living with HIV aged 18–23 years old and 24+ years old	227	February 2017 – March 2017
Namale et al., 2019	5	Sustained virological response and drug resistance among female sex workers living with HIV on antiretroviral therapy in Kampala, Uganda: a cross-sectional study	Kampala	Uganda	Programme/Routine, Cross-sectional study	FSW living with HIV on ART aged 18–54 years	584	January 2015 – December 2016
Tun et al., 2019	21	Community-based antiretroviral therapy (ART) delivery for female sex workers in Tanzania: 6-month ART initiation and adherence	Njombe and Mbeya regions	Tanzania	Research, Other: quasi-experimental prospective cohort study	FSW living with HIV aged 18 years or older	617 total: 309 intervention and 308 comparison	July 2017 – October 2017

Note: Please see the full reference list of this article for more information: https://doi.org/10.4102/sajhivmed.v26i1.1626.

ART, antiretroviral therapy; FSW, female sex workers; HCWs, healthcare workers.

All the articles included were published during January 2019 to March 2024. Nine of the 10 included studies did not include an intervention. However, two articles included an intervention that stemmed from the same study, the first being an article based on the intervention,^21^ while the other was an embedded qualitative study of that intervention.^22^ Three of the 10 studies were conducted in Tanzania. The first study by Tun et al. examined the effectiveness of community-based ART delivery specifically for FSWs in Tanzania, focusing on 6-month ART initiation and adherence.^21^ The second study by Tun et al. explored the perceived high quality of care in community-based ART services for FSWs living with HIV in Tanzania, and investigated the effectiveness and acceptability of the decentralised approach.^22^ Tun et al. investigated the factors that either facilitate or hinder the linkage to HIV care and treatment among FSWs in Tanzania.^22^ The studies conducted in Uganda focused on challenges and successes related to enrolling FSWs in HIV care within the health facilities in Mabarar city in Uganda^24^; factors influencing retention and non-viral suppression among FSWs on ART from primary healthcare facilities^25^; views of FSWs regarding the DSD models for HIV care^26^; and sustained virological response and the prevalence of drug resistance among FSWs on ART.^5^ The study by Vu et al. compared a community-based ART model for FSWs in Malawi with centralised government-run services.^27^ In a study conducted in Bulawayo, Zimbabwe, the author delved into the experiences and challenges faced by FSWs when accessing HIV care services.^28^ In South Africa, the preferences of FSWs living with HIV in Durban regarding community-based decentralised treatment options were explored.^29^

### Types of differentiated service delivery models for HIV treatment among female sex workers in sub-Saharan Africa

Table 4 summarises the types of DSD models indentified in this review, as well as the factors associated with the implementation of these DSD models for HIV treatment among FSWs in SSA. These studies focused on HIV care and treatment among FSWs, with a particular emphasis on improving HIV access, adherence, retention, and outcomes through the various DSD models.

**TABLE 4:** Impact of differentiated service delivery model on HIV treatment outcomes and related factors among female sex workers in sub-Saharan Africa.

Authors, year	Reference	Title	Country	Data type, study design	HIV treatment outcomes	Other related outcomes
Tun et al., 2023	22	Quality of care is perceived to be high with community-based antiretroviral therapy (ART) services for female sex workers in Tanzania: Qualitative findings from a pilot implementation science study	Tanzania	Research, Qualitative study	**Higher initiation:** Community-based intervention significantly increased ART initiation **Improved retention:** Intervention group had higher retention at 12 months **Lower discontinuation:** One intervention participant versus three from the comparison group stopped ART for more than 30 days in the last 6 months **Viral load testing:** Comparison group tested for viral load but did not receive results	Improved access to ART
Arinaitwe et al., 2023	24	Enrollment and retention of female sex workers in HIV care in health facilities in Mbarara City	Uganda	Research, Qualitative study	Improved retention to care Limited access to comprehensive services	
Comins et al., 2022	29	Opportunities and considerations for the design of decentralized delivery of antiretroviral therapy for female sex workers living with HIV in South Africa	South Africa	Research, Qualitative study	**Current ART use:** FSWs currently on ART: 50% (12/24) successfully engaged in treatment **Viral suppression:** Community-based distribution (DTP): higher likelihood of viral suppression among ART patients Tested FSWs: 90.9% achieved viral suppression (≤ 1000 copies/mL), like the general population in Uganda **Retention in care:** **Initial retention:** 85% of newly diagnosed FSWs remained in care at 6 months **Improved retention:** Tailored services like flexible appointments and supportive counselling enhanced retention **Long-term retention:** Declined over time – 74% retained at 24 months **Lost to follow-up:** 26% of FSWs dropped out by 24 months, especially younger FSWs (< 25 years)	
Atuhaire et al., 2022	25	A retrospective cross-sectional study assessing factors associated with retention and non-viral suppression among HIV positive FSWs receiving antiretroviral therapy from primary health care facilities in Kampala, Uganda	Uganda	Programme/ Routine, Cross-sectional study	**FSWs currently on ART:** 50% (12/24) successfully engaged in treatment	
Atuhaire et al.,2022	26	‘My condition is my secret’: perspectives of HIV positive female sex workers on differentiated service delivery models in Kampala Uganda	Uganda	Research, Qualitative study	Retention in HIV care	Access to HIV care
Moyo et al., 2021	28	The experiences of sex workers accessing HIV care services in Bulawayo, Zimbabwe	Zimbabwe	Research, Qualitative study	Higher retention rates	Increased access to HIV services Enhanced knowledge and understanding of HIV treatment and prevention methods
Vu et al., 2021	27	Assessment of community-based ART service model linking female sex workers to HIV care and treatment in Blantyre and Mangochi, Malawi	Malawi	Mixed-method implementation science study (prospective cohort design)	**ART initiation:** Initiation: 100% (DIC) vs. 92% (health facilities) Same-day initiation: 76% (DIC) vs. 55% (health facilities) Current ART use: 85% (DIC) vs. 88% (health facilities) **Retention in care:** ART-naïve: 85% (DIC) vs. 73% (health facilities) Defaulted: 16% (DIC) vs. 27% (health facilities) **Viral load suppression:** Health facilities: 100% virally suppressed (< 1000 copies/mL) Mixed collection (DIC + health facilities): 88% virally suppressed DIC only: 87% virally suppressed **Adherence issues:** Missed doses (past 30 days): 33% (health facility users), 45% (mixed), 47% (DIC only) Stopped ART for 30+ days (past 6 months): 17% (health facility), 4% (mixed), 20% (DIC only) **Viral load documentation:** Documented in health records (past 6 months): 14% (health facility), 28% (mixed), 27% (DIC only)	
Nyato et al., 2019	23	Facilitators and barriers to linkage to HIV care and treatment among female sex workers in a community-based HIV prevention intervention in Tanzania: A qualitative study	Tanzania	Research, Qualitative study	Viral suppression indicating successful treatment adherence Increased CD4 count Improved adherence to ART Viral load rebound Declining CD4 count Drug resistance Poor adherence to ART Increased HIV-related morbidity and mortality Continued HIV transmission: new infections persisted in certain groups, highlighting ongoing transmission concerns	
Namale et al., 2019	5	Sustained virological response and drug resistance among female sex workers living with HIV on antiretroviral therapy in Kampala, Uganda: a cross-sectional study	Uganda	Programme Routine, Cross-sectional study	Virological suppression rate: a relatively high level of virological suppression was observed, with 91% of participants achieving virological suppression (VL < 1000 copies/mL) Adherence to ART: a significant majority (83%) of participants reported being adherent to their ART treatment, taking > 95% of their prescribed doses Low prevalence of virological failure Drug resistance mutations Virological failure for 18–24-year-old FSWs Non-adherence and low CD4+ counts	
Tun et al., 2019	21	Community-based antiretroviral therapy (ART) delivery for female sex workers in Tanzania: 6-month ART initiation and adherence	Tanzania	Research, Other: quasi-experimental prospective cohort study	High ART initiation rates High adherence to ART	Barriers in accessing care Long-term retention in care were not fully addressed

Note: Please see the full reference list of this article for more information: https://doi.org/10.4102/sajhivmed.v26i1.1626.

DIC, drop-in centres; DTP, decentralised treatment provision; FSWs, female sex workers; VL, viral load.

#### Differentiated service delivery types

The community- and facility-based models included a decentralised treatment provision, a strategy that incorporates nurse-led ART care and treatment at select pick-up points in the community.^8^ In another study, community client-led ART delivery and community drug distribution points drop-in centres were utilised^24^ to further implement community-based strategies.^26^ Clinical and structural support services in the community were strategies to improve treatment linkage.^23^

Four articles focused on a facility-based DSD model.^5,24,25,28^ These studies on facility-based models were described as utilising mixed strategies, such as in incorporating individual management and fast-track drug refills in treatment delivery. Two articles included both a community- and a facility-based DSD model.^26,27^ DSD models from two studies were unidentifiable, but appeared to combine services delivered through clinics model categorised as facility-based.

#### Differentiated service delivery outcomes

**Community-based differentiated service delivery model:** A study by Tun et al. found that implementation of community-based treatment delivery has a significant positive impact on FSWs, particularly on ART retention, retention in ART services, and adherence to treatment compared to the comparison group.^22^ FSWs in the community-based ART intervention arm were more likely to be retained in ART services after 12 months, while participants in the comparison group had viral load tests conducted, but they did not receive the results.^22^ A study by Comins et al. found that the community-based DSD model could result in ART adherence and retention, reduced stigma, and enhanced confidentiality, making it a promising approach for FSWs.^8^ Nyato et al. highlighted positive outcomes to clinical and structural support services and treatment in the community, which include increased motivation among FSWs to access services, reduced financial barriers through transport facilitation, and improved perceptions of healthcare providers.^23^

Vu et al.^27^ showed higher rates of ART initiation, facilitated by same-day initiation and the supportive environment of drop-in centres (DICs). FSWs utilising DICs showed higher retention and viral suppression rates, indicating their effectiveness in supporting treatment adherence.

**Health facility-based differentiated service delivery model:** A cross-sectional study by Arinaitwe et al.^24^ found that HCWs and comprehensive HIV care services were identified as crucial factors to FSWs being retained in HIV care at facilities. Key factors including positive HCW’s attitudes, counselling and testing, and ART availability contributed to enrolment and retention.^24^ Community outreach services, especially those involving peer support, were highly effective in enrolling FSWs into HIV care, highlighting the importance of community engagement in healthcare.^24^ Similarly, Moyo et al.^28^ found that the positive and negative perceptions among FSWs towards HCWs played a crucial role in the retention of HIV services in facilities. This study also highlighted the importance of addressing socio-economic factors to improve the quality of care and enhance overall HIV care service delivery among FSWs, particularly in public facilities.^28^

In their retrospective study, Atuhaire et al. highlighted the importance of targeted interventions and strategies to improve retention rates.^25^ The authors indicated the need for careful consideration at critical early stages of implementation, as this study found a high percentage of loss to follow-up among FSWs in the first 24 months.^25^

In a study by Namale et al.,^5^ conducted at the Good Health for Women Project, where services were implemented to provide free care, intensive adherence counselling, and active patient follow-up in the clinic, several observations from this type of DSD were made. The Good Health for Women Project was a clinic accredited by the Ministry of Health in Uganda to provide ART, and started initiating ART to FSWs in January 2013. Among 591 individuals attending the clinic for ART treatment, 432 underwent viral load testing. Of these, 83% remained adherent to treatment, while 9% experienced virological failure, indicating that the services provided in the facility may have influenced the high adherence. Notably, FSWs with no education had a higher virological failure rate (15%) compared to those with secondary education and above (12%). Factors influencing virological failure included age, with a higher rate among younger participants (18% in the 18–24 age group) compared to older individuals (> 35 years, 6%). Non-adherent participants also exhibited a higher virological failure rate (16%) compared to adherent individuals (7%). Overall, 91% of FSWs were virally suppressed.^5^

**Mixed differentiated service delivery model: Comprising facility and community-based models:** A study by Arinaitwe et al.^24^ provides insights indicating that different DSD models cater to the diverse needs and preferences of FSWs, addressing factors such as accessibility, confidentiality, waiting times, and peer support.^24,25,26^ An assessment of a study looking at community-based ART services at DICs in Malawi showed improved health outcomes for the FSWs compared to traditional health facilities, offering same-day initiation, peer support, and a less crowded environment, which were preferred by FSWs.^27^ The two DSD models helped to reduce barriers to healthcare and improve access to ART services FSWs living with HIV.^26^

### Impact of the differentiated service delivery model on HIV treatment outcomes and related factors among female sex workers in sub-Saharan Africa

Nine studies explored the effectiveness of various DSD models for FSWs living with HIV, focusing on HIV treatment outcomes and other related outcomes. A community-based ART study delivery by Tun et al., showed improved ART initiation, retention at 12 months, and reduced ART discontinuation rates.^22^ However, participants in the comparison group did not receive feedback on their viral load test results, potentially impacting adherence.^22^ Similarly, Arinaitwe et al. highlighted improved retention in HIV care for FSWs in Uganda, but noted limited access to comprehensive services beyond HIV care.^24^

In South Africa, Comins et al. found that 50% of FSWs successfully engaged in ART, with 90.9% achieving viral suppression through community-based distribution models. Retention rates remained high initially but dropped over time, with younger FSWs (< 25 years) more likely to discontinue treatment.^8^ A study by Atuhaire et al. similarly reported that 50% of FSWs in Kampala, Uganda, were engaged in ART, though no additional outcomes were discussed.^25^ Another study by Atuhaire et al. emphasised the importance of retention in HIV care, with increased access to care through differentiated models.^26^

Moyo et al. examined the experiences of sex workers in Bulawayo, Zimbabwe, and found higher retention rates, improved access to HIV services, and increased knowledge about HIV treatment and prevention methods.^28^ In Malawi, Vu et al. compared ART initiation and retention between DIC and health facility models, finding higher initiation and retention rates at DICs. Viral suppression rates were also high across both models, although adherence challenges were noted, particularly among those relying solely on DIC services.^27^

In Tanzania, high levels of ART adherence and viral suppression were reported, but concerns were raised regarding viral load rebound, drug resistance, and declining CD4 counts among some FSWs. The study highlighted ongoing HIV transmission concerns despite treatment efforts.^23^ Finally, Namale et al. observed high virological suppression (91%) and ART adherence (83%) in Uganda, although younger FSWs experienced higher rates of virological failure and drug resistance.^5^ Tun et al. also noted high ART initiation and adherence in Tanzania, but highlighted barriers to long-term care retention.^21^ Only Atuhaire et al. did not provide detailed HIV treatment outcomes beyond ART engagement.^25^

### Intervention-based study among female sex workers living with HIV in sub-Saharan Africa

Tun et al. conducted an intervention-based trial in Tanzania utilising two different approaches to providing ART services. A community-based DSD type was applied on both the intervention and comparison arms. The intervention arm included community-based HIV testing and counselling teams (CBHTC+) study, while the comparison arm received services at government-designated ART Care and Treatment Centers (CTC).^21^

In the intervention arm, community-centred care provided services to FSWs in the community (CBHTC+ sites) to be less stigmatising with regard to in-service provision.^21^ This type of DSD allowed drug pick-ups at the client’s convenient and safe space, providing flexible scheduling, and delivering medication supplies in varying durations, demonstrating a patient-centred approach. The training of healthcare providers in ‘test and start’ and community-based ART delivery reflected an adaptation to evolving HIV treatment guidelines. The use of text messaging and WhatsApp for regular contact with peer educators demonstrated an integration of technology into healthcare delivery. This approach leveraged mobile communication for monitoring, support, and education of FSWs.^21^

The DSD model outlined in the intervention arm reflects an individualised patient-centred and community-integrated model for HIV testing and treatment. The focus of the model is centred on reducing stigma, enhancing accessibility, leveraging technology, and providing comprehensive care among FSWs, to address the multifaceted challenges associated with HIV care and support.^21^

### Comparison arm

The comparison arm provided ART services at government CTCs to be structured in its approach to managing HIV/AIDS treatment. Enrolment in care was through CTC sites, suggesting a systematic process for initiating and monitoring treatment.^21^ Within the comparison arm, FSWs received adherence counselling sessions, drug pick-up at government centres, and standard client assessment and referrals to family planning and additional services.^21^ Where this arm differed was particularly in the limited availability of service providers for FSWs in facilities, accessibility to services entailing a longer commute, and more crowded facilities with longer wait times.^21^

### Differentiated service delivery outcomes of intervention

Tun et al. found that the intervention group achieved significantly better outcomes.^21^ All participants (100%) in the intervention group registered for HIV care, compared to 72.7% in the comparison group. Similarly, 100% of participants in the intervention group initiated ART, whereas only 71.5% did so in the comparison group. At the 6-month follow-up, all intervention group participants (100%) were still on ART, compared to 95% in the comparison group. Additionally, only 0.9% of the intervention group had discontinued ART for more than 30 consecutive days, compared to a higher percentage in the comparison group.^21^ Additionally, participants in the intervention arm experienced lower levels of internalised stigma (26.6% vs 39.9%).^21^ The intervention demonstrated positive impacts, including higher rates of HIV care registration, lower internalised stigma, increased ART initiation, better adherence to ART, and a higher completion rate for follow-up visits compared to the comparison arm.

### Enablers and challenges associated with differentiated service delivery models for HIV treatment outcomes among female sex workers in sub-Saharan Africa

**FIGURE 2:**
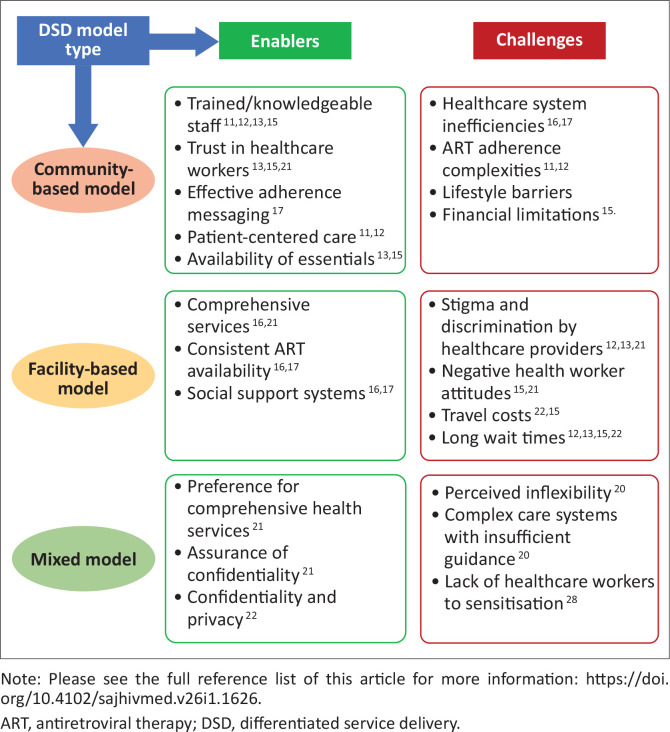
Enablers and challenges in the differentiated service delivery model for HIV treatment outcomes.

Studies reporting on the DSD model for HIV treatment among FSWs highlighted both enablers and challenges (see Figure 2). The success of the community-based care model for FSWs is attributed to a combination of factors, including well-trained staff, effective adherence messaging, satisfaction with diverse services, patient-centred care, trust in HCWs, and the availability of essential services.^13,21,22^ These elements collectively contribute to a positive and supportive healthcare environment for FSWs, enhancing their engagement and adherence to HIV care. When HCWs know how to provide the right care for FSWs, it leads to better health services.^21,22,29^ Patient-centred care means that HIV services are designed around the needs of the individual or clients, which helps them feel more included and satisfied with their care.^22^

Building trust between FSWs and healthcare providers is essential, because clients are more likely to seek help without worrying about stigmatisation. Additionally, access to important services, such as HIV testing and treatment for other infections, makes it easier for FSWs to get the care they need.^8,21,22,24,26,29^ However, there are challenges associated with the use of the community-based care model, such as inefficiencies in the healthcare system that lead to delays in HIV treatment, making it harder for FSWs to receive timely care.^25,26^ Lifestyle factors such as fear of being judged or discrimination can complicate ART adherence. This model entails prolonged pre-enrolment periods and mixed health service delivery, which is associated with confidentiality and privacy issues, perceived health status, and relationship fears.^21^ Financial issues, like the cost of transportation, can also limit access to HIV care by FSWs.^21,22^ Other barriers include a lack of healthcare workers’ sensitisation to sex work and the need for flexible HIV services to accommodate the diverse needs of FSWs.^29^

The facility-based care model has its own advantages and challenges. One strength is that it offers comprehensive services that address many health needs at once, making it easier for FSWs to get the treatment they require. Consistent access to ART is critical because uninterrupted treatment supports better health outcomes.^25^ Additionally, social support systems in these facilities can help FSWs feel less isolated and provide encouragement.^25,26^ Despite these benefits, the facility-based care model can be difficult for FSWs. Stigma and negative attitudes from HCWs can create a hostile environment, preventing FSWs from seeking care.^21,22,23^ Long wait times and travel costs can further hinder access to services, particularly for those who may not have a lot of money or flexible schedules.^21,22,23,24,26,27,28,29^.

Mixed models of care combine elements from both the community- and facility-based models. Many FSWs appreciate the variety of services offered in facility-based models, which help improve their health and increase their comfort level. Ensuring privacy is very important; when healthcare providers prioritise confidentiality, FSWs are more willing to seek help without fear of being exposed or judged.^27^ However, mixed models also face challenges, such as a perception of HIV services not being flexible.^22^ The complexity of healthcare systems and a lack of clear guidance can lead to treatment interruptions, as navigating these systems can be overwhelming.^27^

## Discussion

This systematic review aimed to explore the status quo on the implementation of treatment-focused DSD models in improving HIV treatment outcomes among FSWs in SSA. This review explored various aspects, including decentralised ART delivery, challenges and successes in HIV care, factors influencing retention, DSD models, antiretroviral drug use, long-acting injectable therapy, linkage to HIV care, sustained virological response, and community-based ART delivery.

The included studies revealed insights into the effectiveness and acceptability of decentralised ART services for FSWs, challenges in enrolling and retaining FSWs in HIV care, and the experiences of FSWs in accessing HIV care services. They also examined factors affecting retention and non-viral suppression, FSWs’ viewpoints on service delivery models, and the prevalence of drug resistance. The research highlighted the potential benefits and challenges associated with decentralised ART delivery and provided insights into some model of HIV care for FSWs in SSA.

DSD models for HIV care among FSWs in SSA indicate that successful HIV care for FSWs requires a nuanced and comprehensive approach. The DSD models must be adaptable to local contexts. The 2019 study by Tun et al. provides valuable insights into the effectiveness of a community-centred DSD model in improving HIV care outcomes for FSWs in Tanzania.^21^ A study in Nigeria found that multi-month dispensing and community-based ART were effective strategies for improving ART adherence during the COVID-19 pandemic.^30^ Interestingly, an increase in viral load tests was observed during the pandemic compared to the pre-COVID-19 period, contradicting the findings reported in Uganda.^25^ These results emphasise the importance of flexible, patient-centred approaches, and technological integration in optimising the provision of differentiated services to this key population.^30^

Our findings demonstrate that the community-based model displays positive outcomes through well-trained staff, patient-centred care, and technology integration, while facing challenges such as system inefficiencies and access because of lack of money. The facility-based model succeeds in trust-building, comprehensive services, and consistent ART availability, but encounters multifaceted challenges at both the individual and societal levels. The mixed model combines strengths but faces challenges such as inflexibility, not adapting to the needs of FSWs regarding opening for longer hours, and negative HCW attitudes. The findings emphasise the importance of a nuanced and adaptive approach, community engagement, holistic care, flexibility, technology integration, and addressing systemic challenges for effective and accessible HIV care for FSWs. Although the use of mixed models presents certain challenges, relying solely on private pharmacies has shown promise in playing a key role in differentiated HIV service delivery in SSA. Pharmacies have the potential to reach populations whose HIV prevention and treatment needs are not fully met by conventional clinic-based models. Growing evidence suggests that pharmacy-based HIV services may be both feasible and acceptable in various settings.^31^

### Public health implications

Successful HIV care for FSWs in SSA requires a tailored, adaptable, and community-engaged approach. Ongoing efforts should focus on refining interventions based on identified challenges and successes. Flexibility, patient-centred approaches, and technology integration are pivotal for optimising DSD. Ongoing efforts should focus on refining interventions, addressing socio-economic factors, and fostering community engagement to create sustainable improvements in HIV care outcomes for this key population. The results of the review implicate the need to develop targeted interventions to improve HIV outcomes for this population. The study by Eshun-Wilson et al.^32^ demonstrates that both community-based and facility-based models are effective in retaining patients in care, and are generally acceptable to patients in the short to medium term. The authors believe that, while broad public health strategies are essential for expanding access to ART, it is also important to incorporate elements of patient choice into the model design, whenever feasible.

The findings from this review could guide HIV policymakers on the most effective strategies for implementing DSD models across various settings and improving HIV outcomes for FSWs living with HIV, including enhancing their quality of care. Applying the DSD model to this population will promote the efficient operation of the healthcare system, enabling it to allocate resources to those with the greatest need.

### Strengths

A strength of this study is its emphasis on a key population, namely FSWs living with HIV in SSA. By targeting this specific group, the research sheds light on the distinct challenges and successes related to the implementation of DSD models for HIV care. Additionally, the comprehensive nature of the study, which evaluates various DSD models, such as community-based, facility-based, mixed approaches, and the emerging role of private pharmacies, enables a well-rounded understanding of how decentralised ART delivery can enhance outcomes for FSWs living with HIV. The inclusion of research from multiple countries across SSA further enriches the geographic relevance, offering insights into how DSD models perform within different social and healthcare environments. Another notable strength is the study’s relevance, particularly as it incorporates evidence from the COVID-19 pandemic, showcasing how multi-month dispensing and community-based ART initiatives supported adherence during this challenging period. The focus on decentralised care is crucial for populations that encounter stigma and logistical obstacles, reinforcing the practical significance of the findings for improving HIV treatment access among key populations. By integrating studies that emphasise patient-centred approaches, the review highlights flexible and adaptable models that can be implemented across various contexts in SSA.

### Limitations

Despite its strengths, the study does have limitations. One of the main drawbacks is the limited availability of high-quality research specifically addressing FSWs living with HIV, which may have constrained the depth of the analysis. The decision to exclude non-English studies may also have narrowed the scope of the review, potentially overlooking important research from French- or Portuguese-speaking regions. This linguistic bias could limit the applicability of the findings, as different cultural and healthcare contexts might yield varying outcomes for the DSD models. Furthermore, the diversity of study designs and methodologies complicated direct comparisons of results, which may hinder the ability to draw conclusive insights. Additionally, the focus on short-term outcomes, such as ART adherence and retention, creates gaps in understanding the long-term effectiveness of DSD models, including sustained virological suppression and overall quality of life. While the review does address systemic inefficiencies and financial barriers, it falls short of thoroughly exploring broader structural factors such as legal challenges, societal stigma, and human rights issues that are critical barriers for FSWs living with HIV. Lastly there is a possibility of publication bias, as studies reporting positive outcomes tend to be published more often, which could lead to an overrepresentation of successful DSD models while underreporting their limitations or failures.

## Conclusion

This review provides a comprehensive overview of the DSD models for HIV care among FSWs in SSA. It shows how different DSD approaches effectively improve HIV treatment for FSWs. The results highlight the importance of implementing targeted interventions that consider the intricate interaction of personal, societal, and structural factors to improve HIV outcomes. Overall, the review demonstrates the positive impact of DSD models, including community-based, facility-based, and mixed approaches, in improving ART initiation, retention, adherence, and viral suppression among FSWs. Community-based models offer promising outcomes, but face challenges related to healthcare system inefficiencies and financial barriers. Facility-based models emphasise trust in HCWs and provide comprehensive services, but encounter barriers such as long wait times and transportation costs. Mixed models provide flexible and comprehensive care but require a nuanced approach to address challenges such as perceived inflexibility and trust issues.

The COVID-19 pandemic has accelerated the adoption of the DSD models, highlighting the importance of integrating community-based services and virtual platforms into healthcare systems. Moving forward, it is crucial to address systemic challenges to ensure the successful implementation of the DSD models and provide high-quality, person-centred care for FSWs living with HIV. Tailored interventions, context-specific approaches, and ongoing efforts to address socio-economic factors are essential to optimise HIV care delivery for this key population across diverse settings in SSA and beyond.
